# Involvement of five catalytically active Arabidopsis β‐amylases in leaf starch metabolism and plant growth

**DOI:** 10.1002/pld3.199

**Published:** 2020-02-11

**Authors:** Jonathan D. Monroe

**Affiliations:** ^1^ Department of Biology James Madison University Harrisonburg Virginia

**Keywords:** cold stress, osmotic stress, starch, starch degradation, β‐amylase

## Abstract

Starch degradation in chloroplasts requires β‐amylase (BAM) activity, but in Arabidopsis, there are nine BAM proteins, five of which are thought to be catalytic. Although single‐gene knockouts revealed the necessity of BAM3 for starch degradation, contributions of other BAMs are poorly understood. Moreover, it is not possible to detect the contribution of individual BAMs in plants containing multiple active BAMs. Therefore, we constructed a set of five quadruple mutants each expressing only one catalytically active BAM, and a quintuple mutant missing all of these BAMs (*B‐Null*). Using these mutants, we assessed the influence of each individual BAM on plant growth and on leaf starch degradation. Both BAM1 and BAM3 alone support wild‐type (WT) levels of growth. BAM3 alone is sufficient to degrade leaf starch completely whereas BAM1 alone can only partially degrade leaf starch. In contrast, BAM2, BAM5, and BAM6 have no detectable effect on starch degradation or plant growth, being comparable with the *B‐Null* plants. *B‐Null* plant extracts contained no measurable amylase activity, whereas BAM3 and BAM1 contributed about 70% and 14% of the WT activity, respectively. BAM2 activity was low but detectable and BAM6 contributed no measurable activity. Interestingly, activity of BAM1 and BAM3 in the mutants varied little developmentally or diurnally, and did not increase appreciably in response to osmotic or cold stress. With these genetic lines, we now have new opportunities to investigate members of this diverse gene family.

## 1 INTRODUCTION

Starch is the storage form of energy and reduced carbon in most plants, normally accumulating during the day as large granules in chloroplasts of green tissues (transitory starch), and over a season in amyloplasts of seeds and storage tissues (storage starch). Both types of starch granules contain two polymers: amylopectin and amylose, both of which are composed of α‐1,4‐linked glucose. Amylopectin also contains α‐1,6‐linked branches and makes up about 80% of the granule whereas the remainder, amylose, is largely unbranched. Analysis of Arabidopsis mutants has shed much light on our understanding of starch metabolism (Pfister & Zeeman, 2016; Zeeman, Kossmann, & Smith, 2010), but details are still emerging such as the specific roles of relevant gene family members.

Starch is synthesized from ADP–glucose by a family of starch synthases (soluble and granule‐bound) that generate linear α‐1,4‐linked glucan chains (Zeeman et al., 2010). Starch branching enzymes (BEs) then introduce α‐1,6‐branches (Tomlinson & Denyer, 2003), some of which are removed by isoamylase (ISA1 and ISA2) (Delatte, Trevisan, Parker, & Zeeman, 2005) resulting in the formation of layers in which regions of branches are interspersed with crystalline regions composed of short (12–15 residue), α‐glucan segments that form double helices (Buléon, Colonna, Planchot, & Ball, 1998). A consequence of this pattern of layers is that granules can become extremely large, commonly over 2,000 times the diameter of glycogen granules, the glucose storage polymer in animals (Ball, Colleoni, Cenci, Raj, & Tirtiaux, 2011). Tight packing of the double helices also excludes water making starch a good storage polymer because it lacks osmotic activity.

Starch degradation in Arabidopsis leaves begins with phosphorylation of the outer chains by two water dikinases (GWD1 and PWD) that phosphorylate glucose at the C6 and C3 positions, respectively (Baunsgaard et al., 2005; Kötting et al., 2005; Ritte et al., 2002). This phosphorylation is thought to disrupt the double‐helical structure of the outer chains facilitating hydration and subsequent hydrolysis, primarily by BAM3 and BAM1, two of the nine β‐amylases (http://www.chem.qmul.ac.uk/iubmb/enzyme/EC:3/2/1/2.html; CAZy Family 14) in Arabidopsis (Fulton et al., 2008). Maltose released by the BAMs is exported from chloroplasts by the MEX1 transporter for further metabolism in the cytosol (Nittylä et al., 2004). Prior to complete hydrolysis of the outer chains, phosphates are removed by two phosphoglucan phosphatases (SEX4 and LSF2) (Kötting et al., 2009; Santelia et al., 2011). Third, catalytically inactive phosphoglucan phosphatase‐like protein, LSF1, acts as a scaffold binding BAM3 and BAM1 (Schreier et al., 2019). Complete degradation of starch also requires hydrolysis of the α‐1,6‐branches, which is carried out by a different isoamylase (ISA3), and limit dextrinase (LDA) (Streb & Zeeman 2012). A plastid‐localized α‐amylase (AMY3) may also play a role in starch degradation at the granule surface (Seung et al., 2013).

In Arabidopsis, there are nine BAM genes that were recently reviewed (Monroe & Storm 2018; Thalmann et al., 2019). Two of these genes, *BAM7* and ‐*8*, encode proteins with N‐terminal DNA‐binding domains that are targeted to nuclei where they function in regulating gene expression (Reinhold et al., 2011; Soyk et al., 2014). The Arabidopsis forms of BAM7 and ‐8 have no apparent catalytic activity as β‐amylases. One of the other BAM genes, *BAM4*, encodes a catalytically inactive protein located in plastids where it may play a role in regulating starch metabolism (Fulton et al., 2008; Li et al., 2009). Evidence suggests that BAM9 may also be plastidic and catalytically inactive (Monroe & Storm, 2018). Comparison of active‐site residues (Laederach, Dowd, Coutinho, & Reilly, 1999) in these four BAM proteins with those from catalytically active BAMs reveals differences that are consistent with their lack of catalytic activity (Monroe & Storm, 2018).

Of the five remaining BAM genes, four (*BAM1*, ‐*2*, ‐*3*, and ‐*5*) encode enzymes that are known to be catalytically active (Monroe & Preiss 1990; Monroe et al., 2017; Fulton et al., 2008; Lao et al., 1999; Li et al., 2009; Sparla, Costa, Schiavo, Pupillo, & Trost, 2006) and one (*BAM6*) encodes a protein that is predicted to be active based on its amino acid sequence (Monroe & Storm, 2018). Of these five BAM proteins, three (BAM1, ‐2, and ‐3) were shown to be located in plastids where they could participate directly in starch degradation (Fulton et al., 2008; Lao et al., 1999). BAM6 has a predicted chloroplast transit peptide and was detected in the chloroplast proteome (Zybailov et al., 2008). The only catalytically active BAM that is not located in plastids is BAM5, which is a cytosolic enzyme found in phloem tissue where it is unlikely to come into contact with plastidic starch (Monroe & Preiss 1990; Laby, Kim, & Gibson, 2001; Wang, Monroe, & Sjolund, 1995). The function of BAM5 is currently unknown.

Studies using mutants in which individual genes are knocked‐out or defective have been extremely useful in determining the function of some genes, but other mutants lack phenotypes in part due to genetic redundancy (Bouché & Bouchez, 2001). For example, single mutants revealed that transitory starch accumulated in the mutant lacking BAM3 but not in the mutant lacking BAM1, yet both genes are known to play a role in this process (Fulton et al., 2008). Understanding the effects of single‐gene mutations is especially difficult for those genes that encode enzymes because tissue extracts often contain multiple gene products having similar catalytic activities. One solution to these problems is to generate multiple‐gene knockouts in which only one member of a gene family is functional. These higher‐order mutants can then be compared with mutants lacking all functionally similar members of the family to observe phenotypes associated with the presence of one functional gene as opposed to phenotypes associated with the absence of that gene.

We applied this approach to the β‐amylase gene family in Arabidopsis and present results showing the influence of each of the five catalytically active BAMs on leaf starch accumulation and plant growth. In addition, we measured the catalytic activity of each BAM in above‐ground tissues. Lastly, we determined the effects on BAM1 and BAM3 activity of developmental age, time of day, and various abiotic stresses.

## 2 MATERIALS AND METHODS

### 2.1 Plant material and growth conditions

Arabidopsis (*Arabidopsis thaliana*) ecotype Columbia‐0 plants were grown at 22°C with a 12‐hr‐light/12‐hr‐dark photoperiod and 130 mmol m^−2^ s^−1^ of illumination on a growth cart (Grower's Supply Co) or in a growth chamber (AR‐22L; Percival). The growth medium was ProMix BX (Premier Tech Horticulture) supplemented with macronutrients and micronutrients as described by LEHLE SEEDS. T‐DNA lines were obtained from the Arabidopsis Biological Resource Center and included *bam1* (Salk_039895), *bam2* (Salk_086084), *bam5* (Salk_004259), and *bam6* (Salk_023637). Seeds of *bam3* were a gift from David Seung. For osmotic stress experiments, 200 ml of the same nutrient solution with or without 300 mM mannitol was applied to each pot. For cold stress experiments, plants were transferred to a walk‐in 4°C chamber with lighting as described above.

### 2.2 T‐DNA mutant analysis

T‐DNA lines were verified by PCR using the primers listed in Table S1. The *bam3* mutation was verified using PCR followed by digestion of the PCR product with *Bsr*I. Multiple mutants were generated by crossing homozygous single mutants and allowing self‐pollination of confirmed double heterozygotes.

### 2.3 Starch analysis, enzyme extraction, and assays

Above‐ground parts of plants were harvested and frozen at −80°C for later analysis. The largest 3 to 5 leaves from at least 2 plants were decolorized in hot 80% ethanol and then stained with Lugol iodine solution (Hostettler et al., 2011). Images of representative leaves were collected using a Nikon D7000 camera. For enzyme extraction, tissues were ground in 3 volumes of extraction buffer (50 mM MOPS, pH 7, 5 mM EDTA, and 2 mM dithiothreitol) with sand and centrifuged at 10,000 *g* for 10 min at 4°C. Amylase assays were conducted as described (Monroe et al., 2014), except soluble starch (Acros Organics #424491000) was used as the substrate. Concentrations of soluble starch used in each assay are listed in the text. Assays of extracts from plants that contained WT *BAM2* also included 100 mM KCl. Total reducing sugars were measured using the same assay except soluble starch was omitted. Native, starch‐containing PAGE was conducted as described by Doyle, Lane, Sides, Mudgett, and Monroe (2007). Total protein was measured using the Bio‐Rad Protein Assay Kit with bovine serum albumin as the standard. Means of replicate enzyme assays were analyzed for statistical significance using a two‐tailed Student's *t* test.

## 3 RESULTS

Starch degradation in leaves depends on β‐amylase (BAM) activity, but it is unclear which of the Arabidopsis genes that encode catalytically active BAMs play a role in this process. Analysis of single‐gene knockouts has not revealed phenotypes for some of these genes. We therefore constructed a set of multiple‐gene knockouts, each containing a single active BAM, and compared them with a mutant containing no active BAMs in order to test their effect on growth and starch degradation, and to examine their activity in leaf extracts.

**Figure 1:**
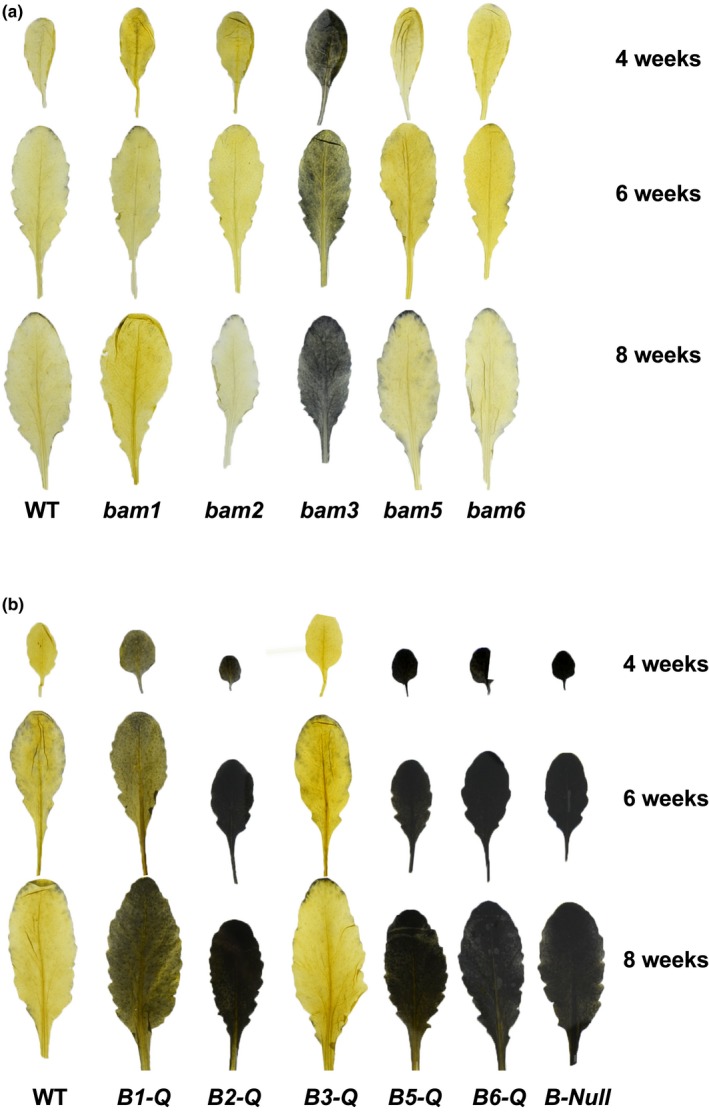
Starch content in leaves of 4‐, 6‐, and 8‐week‐old wild‐type (WT) and mutant Arabidopsis plants. Plants were grown under a 12/12 hr‐light/dark photoperiod, harvested at the end of the night period and stained with iodine. (a) WT and single BAM mutants. (b) WT and quadruple mutants lacking all but one BAM gene, and *B‐Null* lacking all five BAM genes. For each treatment, 3–5 leaves from at least two different plants were analyzed and representative leaves are shown

Mutants of Arabidopsis used in the present work include T‐DNA insertion mutants lacking BAM1 (At3g23920; SALK_039895) and BAM2 (At4g00490; SALK_086084) that were previously characterized and shown to lack the respective mRNAs (Fulton et al., 2008; Kaplan & Guy, 2005). In addition, the mutant lacking BAM3 (At4g17090; CS92461) is a nonsense point mutation in the 4th exon that lacks soluble BAM3 protein (Fulton et al., 2008). The BAM5 mutant (At4g15210; SALK_004259) contains a T‐DNA insertion in the first intron that leads to a lack of detectable BAM5 enzyme activity on a native, starch‐containing gel (Figure S1). The BAM6 mutant (At2g32290; SALK_023637) contains a T‐DNA insertion in the third exon. This mutant was previously shown to have a mild starch excess (*sex*) phenotype in leaves from 8‐week‐old plants (Monroe et al., 2014). The five single BAM mutants were first compared with wild‐type (WT) plants, all grown under a 12‐hr‐light/12‐hr‐dark photoperiod, by iodine staining leaves that were harvested at the end of the night. Only the *bam3* mutant revealed a *sex* phenotype, and this phenotype was similar in leaves harvested from 4‐, 6‐, and 8‐week‐old plants (Figure 1a). None of the other mutants revealed an obvious leaf *sex* phenotype at these ages.

**Figure 2:**
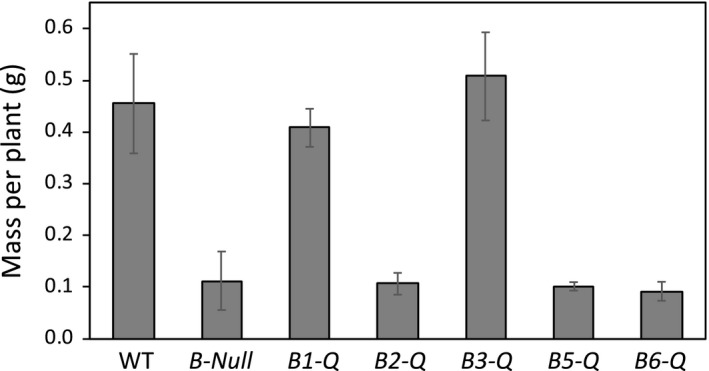
Effect of individual BAM genes on plant mass at 5 weeks of age when grown under a 12‐hr‐light/12‐hr‐dark photoperiod. Data are means ± *SD* of the six largest plants from two pots per genotype. WT, *B1‐Q,* and *B3‐Q* means are not significantly different from each other, as are the remaining four genotypes, but the two groups are significantly different (*p* < .01)

The five mutants each lacking one catalytically active BAM were then crossed multiple times in order to generate five quadruple mutants, each expressing only one catalytically active BAM, and the quintuple mutant lacking all five BAMs. For simplicity, the mutants were named *B1‐Q* for the *bam2*/*bam3*/*bam5*/*bam6* quadruple mutant, *B2‐Q*, etc., and *B‐Null* for the quintuple mutant lacking all five catalytically active BAMs. Mutant alleles were detected by PCR analysis of DNA isolated from leaf tissue (Figure S2). The quadruple mutant containing only BAM3 (*B3‐Q*) was indistinguishable from WT plants in terms of leaf starch content at the end of the night estimated by iodine staining of leaves from 4‐, 6‐, and 8‐week‐old plants (Figure 1b), and plant mass at 5 weeks of age (Figure 2). *B1‐Q* plants had a mild *sex* phenotype similar to that of *bam3* (Figure 1a,b) but were also indistinguishable from WT plants in their mass at 5 weeks of age (Figure 2).

**Figure 3:**
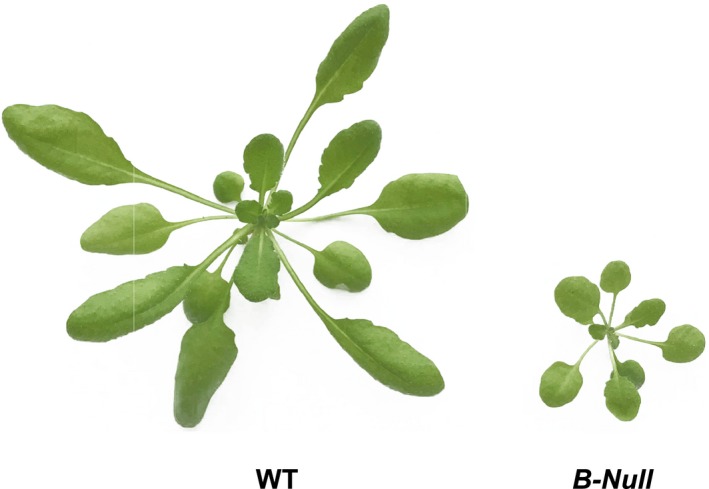
Appearance of WT and *B‐Null* plants at 5 weeks of age. The *B‐Null* plant contains mutations in *BAM1*, ‐*2*, ‐*3*, ‐*5*, *and *‐*6* genes

In contrast to the *B1‐Q* and *B3‐Q* quadruple mutants, the *B‐Null* plants lacking all five catalytically active BAMs accumulated higher levels of leaf starch (Figure 1b) and grew more slowly than WT plants, reaching about 25% of the WT size at 5 weeks of age (Figure 2). Besides being small, *B‐Null* plants appeared normal (Figure 3). *B2‐Q*, *B5‐Q,* and *B6‐Q* plants were all indistinguishable from *B‐Null* plants in terms of leaf starch accumulation, as detected by iodine staining, and size at 5 weeks of age (Figures 1b and 2). Interestingly, each of the mutants germinated at the same rate as the WT and grew normally for the first few days, consistent with Arabidopsis seeds storing lipids and not starch, then the mutants lacking both BAM1 and BAM3 slowed their rate of growth (data not shown) displaying the largest differences at about 5 weeks of age. However, each of the mutants eventually became full sized and produced normal levels of seeds.

**Figure 4:**
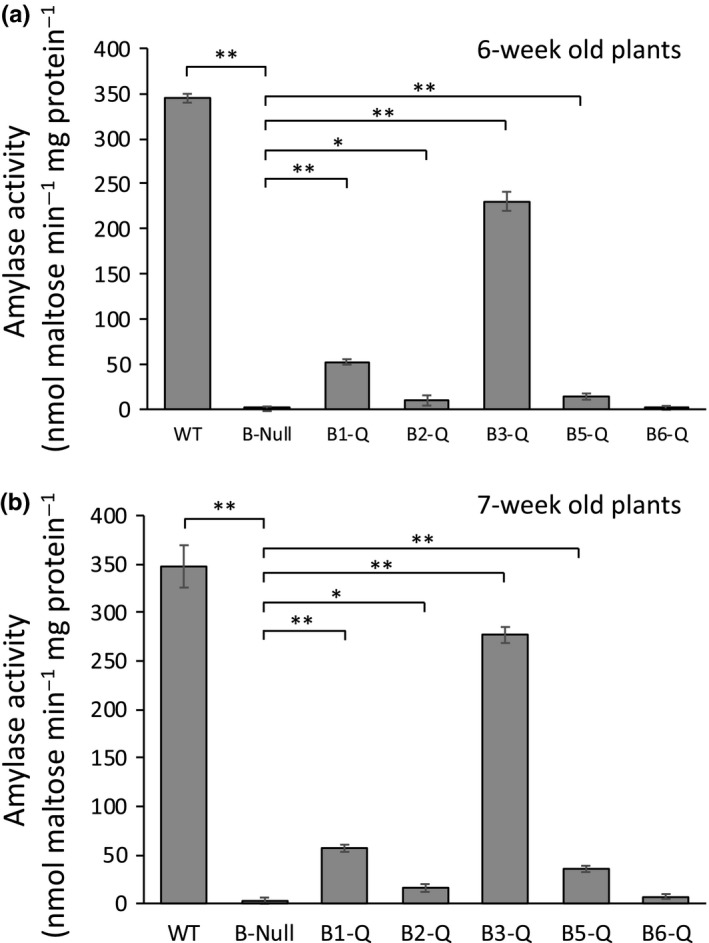
Total amylase activity in crude extracts from leaves of WT and mutant lines of Arabidopsis grown under a 12‐hr‐light/12‐hr‐dark photoperiod. All extracts were assayed at 25℃ in 50 mM MES buffer, pH 6, with 80 mg/ml soluble starch and 100 mM KCl. (a) Plants harvested at 6 weeks of age. (b) Plants harvested at 7 weeks of age. Values are means ± *SD* (*n* = 3). Means that were significantly different from the *B‐Null* activity are labeled with *p < .05, **p < .01

Leaf extracts from WT plants may contain up to four or more different BAM enzymes with similar catalytic activity, so quantifying the activity of individual BAMs in crude extracts is not possible. The quadruple mutants that each contain only one catalytically active BAM offer a solution to this problem. Amylase activity in crude extracts of the quadruple and quintuple mutants was measured at 6 and 7 weeks of age using conditions that are likely to be nearly optimal for four of the five BAMs that have been characterized (Monroe et al., 2014, 2017). Assays were conducted at 25°C and included 80 mg/ml soluble starch, which is near the Vmax for BAM2 and saturating for BAM1, ‐3, and ‐5. Assays also included 100 mM KCl, which is required for BAM2 activity (Monroe et al., 2017). Reducing sugars generated by BAM activity were measured using the Somogyi–Nelson method (Nelson, 1944). Compared with WT leaf extracts, which contained activity measured at about 350 nmol maltose min^−1^ mg protein^−1^, *B‐Null* leaf extracts contained no detectable BAM activity at either age measured (Figure 4). Of the quadruple mutants, *B3‐Q* extracts contained the most activity, which was about 70% of the WT activity. In decreasing order, *B1‐Q* extracts contained about 14% of the WT activity, whereas *B5‐Q* and *B2‐Q* contained 9% and 4% of the WT activity, respectively. All of these activities were significantly higher than activity in *B‐Null* extracts. In contrast, activity in *B6‐Q* was not significantly different than that of *B‐Null* (Figure 4). Amylase activity in each of the mutants was very similar between 6‐ and 7‐week‐old plants and, importantly, the sum of activities in the five quadruple mutants was similar to that of the corresponding WT activity suggesting that the abundance of each enzyme was probably not strongly affected by the absence of the other four enzymes, but this possibility cannot be ruled out.

**Figure 5:**
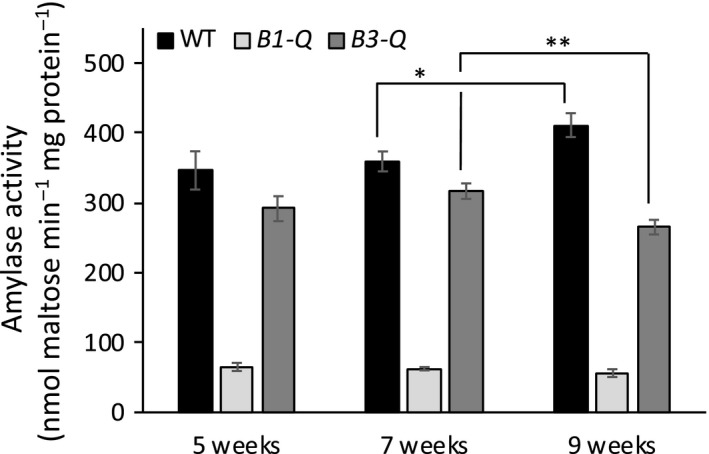
Effect of developmental age on amylase activity in crude extracts from leaves of WT, *B1‐Q* and *B3‐Q* plants grown under a 12‐hr‐light/12‐hr‐dark photoperiod. All extracts were assayed at 25℃ in 50 mM MES buffer, pH 6, using 40 mg/ml soluble starch. Values are means ± *SD* (*n* = 3). Means that were significantly different between weeks are labeled with *p < .05, **p < .01

**Figure 6:**
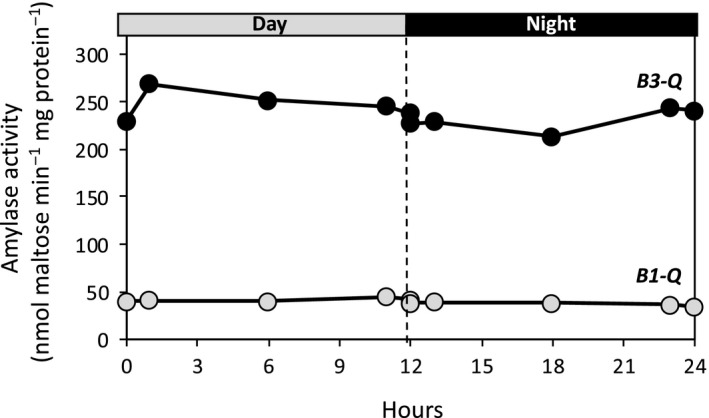
Total amylase activity in crude extracts from leaves of 5‐week‐old *B3‐Q* and *B1‐Q* plants over a diurnal period. Extracts were assayed at 25℃ in 50 mM MES buffer, pH 6, with 40 mg/ml soluble starch. Each point represents the activity in one extract prepared from leaves of 3 plants

It appears that of the five catalytically active BAMs, only BAM3 and BAM1 have a strong influence on leaf starch degradation and plant growth. We then went on to use the *B3‐Q* and *B1‐Q* mutants to examine the effects on BAM3 and BAM1 activity, respectively, of various conditions that are known to influence their mRNA levels. Activity assays and mass spectrometry analysis of protein abundance have led to a general understanding that levels of metabolic enzymes often change very little diurnally or after brief periods of environmental perturbation, despite large changes in the levels of their transcripts (Piques et al., 2009; Skeffington, Graf, Duxbury, Gruissem, & Smith, 2014). It has been well documented that levels of BAM1 and BAM3 transcripts are strongly affected diurnally and by abiotic stress (Smith et al., 2004; Thalmann & Santelia, 2017). To determine whether there were changes in the activity of BAM1 and BAM3 as plants developed, amylase activity in *B1‐Q* and *B3‐Q* plants was compared with activity in WT plants at 5, 7, and 9 weeks of age. Assays were conducted using 40 mg/ml soluble starch, which is nearly saturating for both enzymes (Monroe et al., 2017). BAM3 activity declined slightly in the oldest plants whereas activity in the WT extracts increased slightly, but BAM1 activity did not change significantly (Figure 5). Previously, levels of *BAM1* and *−3* mRNA were shown to vary considerably over a diurnal period with peaks at the night–day and/or day–night transition in 4‐ and 5‐week‐old plants (Figures S3a and b) (Bläsing et al., 2005; Smith et al., 2004). To determine whether BAM1 and ‐3 activity fluctuated diurnally, leaves from *B1‐Q* and *B3‐Q* plants were harvested at various times during the day and night. Neither BAM3 nor BAM1 activity fluctuated dramatically over the 24‐hr period (Figure 6). The absence of one or more BAMs in the quadruple mutants could have influenced the expression of the remaining BAM, so these activity results should be viewed with caution. However, because the sum of the activities in extracts from the five quadruple mutants was similar to that of the WT extract, it is not likely that there were strong pleiotropic effects.

**Figure 7:**
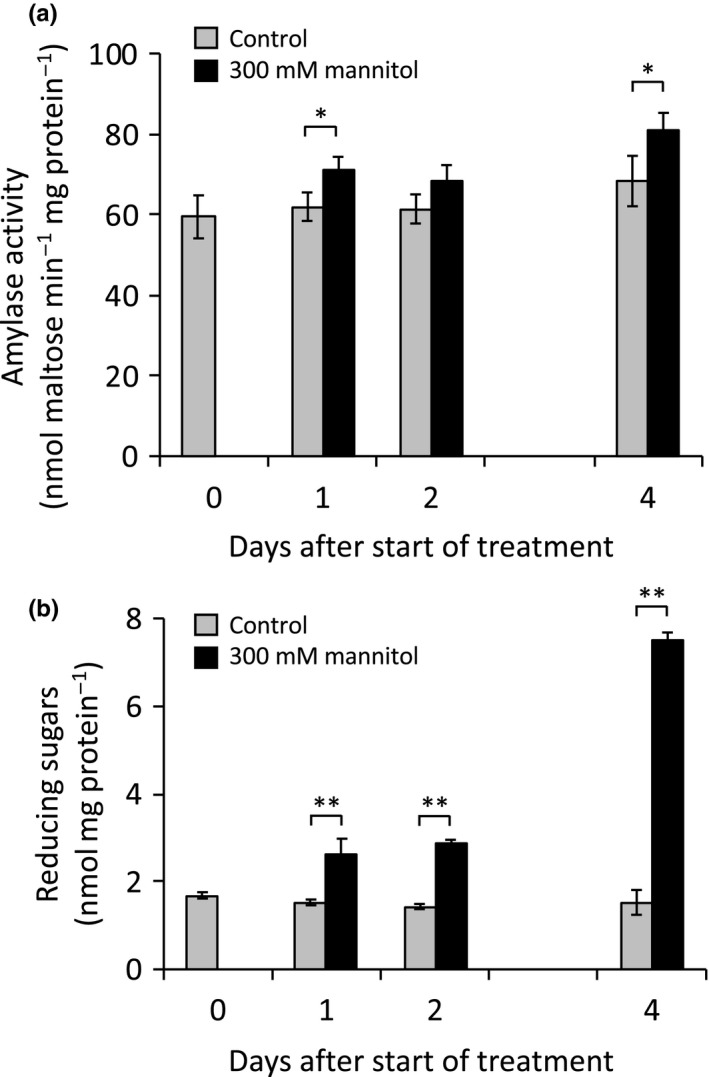
Effect of 300 mM mannitol applied as a soil drench on day zero on amylase activity and reducing sugar levels in *B1‐Q* plants. (a) Amylase activity in leaf extracts assayed at 25℃ in 50 mM MES buffer, pH 6, with 40 mg/ml soluble starch. (b) Reducing sugar levels in the same extracts used in a. Values are means ± *SD* (*n* = 3). Means that were significantly different from the controls of that time point are labeled with *p < .05, **p < .01

**Figure 8:**
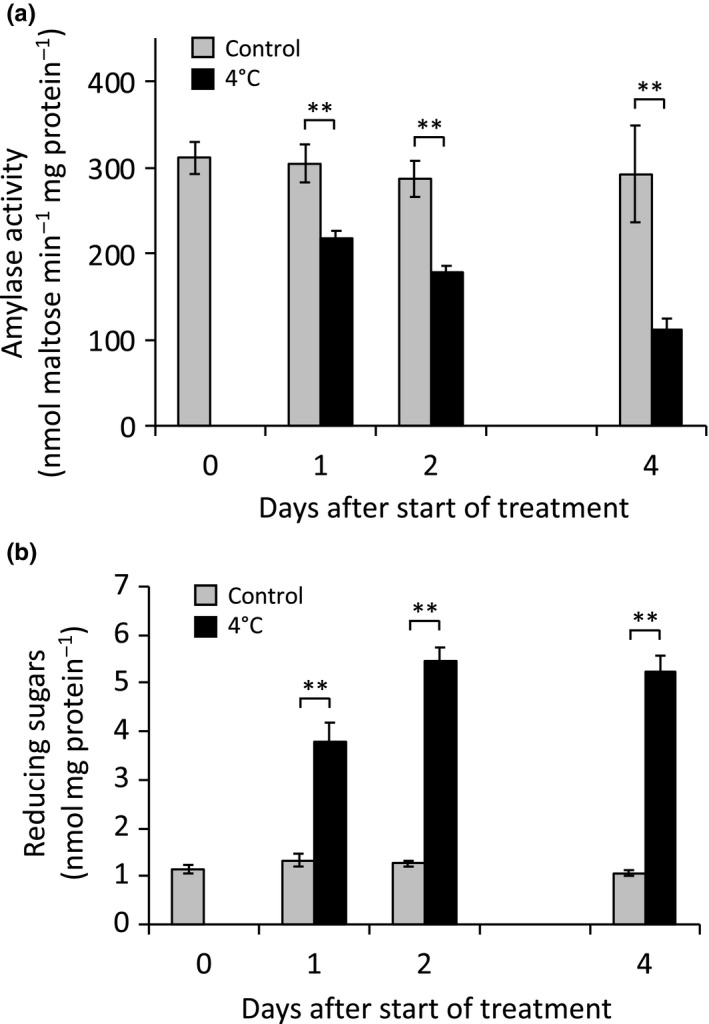
Effect of 4°C cold treatment for up to 4 days on amylase activity and reducing sugar levels in *B3‐Q* plants. (a) Amylase activity in leaf extracts assayed at 25℃ in 50 mM MES buffer, pH 6, using 40 mg/ml soluble starch. (b) Reducing sugar levels in the same extracts used in a. Values are means ± *SD* (*n* = 3). Means that were significantly different from the controls of that time point are labeled with **p < .01

*BAM1* and *BAM3* mRNA levels in Arabidopsis leaves are reported to be strongly influenced by cold and osmotic stress, respectively (see Thalmann & Santelia, 2017 for references). For example, after 24 hr of exposure to 300 mM mannitol, the level of *BAM1* transcript in 18‐day‐old Arabidopsis plants increased 7.6‐fold (Figure S4a), and after 24 hr of 4°C treatment, the level of *BAM3* transcript increased 8.2‐fold (Figure S4b) [data from Kilian et al., (2007) as reported on the Arabidopsis eFP Browser; http://bar.utoronto.ca/efp/cgi-bin/efpWeb.cgi]. We exposed 6‐week‐old *B1‐Q* and *B3‐Q* plants to similar treatments and measured the level of BAM1 and BAM3 activities at 1, 2, and 4 days after the start of treatment. In contrast to the large increase in BAM1 transcript resulting from osmotic stress, BAM1 activity in mannitol‐treated *B1‐Q* plants increased by only 10%–20% compared with control plants over the four days of treatment (Figure 7a). Reducing sugar levels increased by about twofold after 2 days, and by almost fivefold after 4 days of treatment (Figure 7b). In contrast to the effect of cold stress on BAM3 transcript levels, a 4°C cold stress led to a significant decline in BAM3 activity that became stronger over the course of the experiment, decreasing to about 40% of the control levels (Figure 8a). The level of soluble sugars in the cold‐stressed plants increased by over twofold after 1 day and by almost fivefold after 2 days compared with control plants (Figure 8b). Activity of both enzymes and sugar levels in control plants did not change significantly over the course of the experiment.

## 4 DISCUSSION

Generating mutants of Arabidopsis with lesions in multiple members of a gene family can be useful for determining how their expression changes developmentally or physiologically, and ultimately for determining their function. We generated a set of five quadruple mutants of Arabidopsis (*B1‐Q*, *B2‐Q*, *B3‐Q*, *B5‐Q*, and *B6‐*Q), each containing only one of the five potentially catalytically active β‐amylases and one mutant (*B‐Null*) lacking all five BAMs in order to examine their contribution to starch metabolism and growth. We reasoned that the quadruple mutants might also be useful for measuring the activity of individual BAMs in plant extracts, assuming that the extracts contain no other enzymes with similar catalytic activity and that the lack of any given BAM does not strongly influence the expression of other BAMs. *B‐Null* plants contained no detectable BAM activity under the conditions of the assay indicating that there are likely no additional genes in Arabidopsis that encode BAM activity (Figure 4). To minimize the contribution of α‐amylases to the measured activity, we used 5 mM EDTA in the extraction buffer to chelate Ca^+2^, a requirement for some α‐amylases (Swain & Dekker, 1966; Ziegler, 1988). The lack of any detectable amylase activity in the *B‐Null* extracts suggests that α‐amylase activity did not confound the results from other plants.

Leaf starch accumulation (starch excess or *sex*) as determined by iodine staining, although not quantitative, is often used to indicate the involvement of an enzyme in starch degradation, and among the single BAM mutants, only plants without BAM3 activity have this phenotype (Figure 1a). Others have reported similar results (Fulton et al., 2008; Kaplan & Guy, 2005). The lack of a *sex* phenotype in the remaining four catalytically active BAMs (Figure 1a) could indicate that they play no role in starch degradation, or that their phenotype is masked by the activity of a different BAM. Fulton et al. (2008) showed that the double mutant lacking BAM1 and BAM3 had a stronger *sex* phenotype than the single *bam3* mutant illustrating that these two BAMs have overlapping functions and that BAM1 can contribute to starch degradation, but this phenotype is only apparent in the absence of BAM3. Their double mutant contained WT levels of BAM2 and BAM6, the activity of which may therefore be masked by BAM3 and/or BAM1 so it is not possible to evaluate the role of BAM2 or BAM6 in starch metabolism or growth using double mutants.

Compared with *B‐Null* plants lacking all five BAMs, which accumulated high levels of starch and had a severe growth penalty (Figures 2 and 3), the presence of BAM2, BAM5, or BAM6 in *B2‐Q*, *B5‐Q,* and *B6‐Q* plants, respectively, had no observable effect on starch degradation or plant growth (Figures 2 and 3). Small differences in starch levels between the mutants may have been masked by the dark iodine staining and might be detectable using quantitative assays. It will be interesting to determine whether the *B‐Null* plants turn over any starch on a diurnal basis. For BAM5 in *B5‐Q,* the lack of an effect on growth and starch levels was expected because BAM5 is a cytosolic enzyme expressed in phloem tissue, and should therefore have no direct effect on leaf starch degradation (Laby et al., 2001; Wang et al., 1995). The lack of any observable effect of BAM2 and BAM6 on plant growth as compared with *B‐Null* suggests that they do not contribute significantly to leaf starch degradation at the plant ages examined. BAM2 has highly unusual structural and catalytic properties (Monroe et al., 2017; Monroe & Storm 2018) suggesting that it may have a unique role in starch metabolism, and the results reported here suggest that its role does not overlap with the function of BAM3 or BAM1. Activity of BAM2 in assays of *B2‐Q* extracts was low compared with BAM3 activity in *B3‐Q* extracts, but they were significantly higher than that of *B‐Null* (Figure 4). BAM2 activity has only been characterized using the purified enzyme (Monroe et al., 2017; Monroe & Storm 2018), so this is the first evidence that the enzyme is present in shoot extracts. In contrast, activity in *B6‐Q* extracts was not different than that of *B‐Null* extracts so it is possible that the BAM6 protein is not present in above‐ground parts of plants despite evidence of *BAM6* mRNA in leaves (Winter et al., 2007). It is also possible that BAM6 has no catalytic activity despite having all of the conserved, active‐site residues that are consistent with catalytic activity (Monroe & Storm, 2018). A *sex* phenotype was observed in 8‐week‐old *bam6* and *bam3/bam6* plants (Monroe et al., 2014), so BAM6 may only function in older plants. The *BAM6* gene also appears to be restricted to the Brassicaceae (Monroe & Storm, 2018), so it may have a function unique to that family of plants. It is also possible that the natural glucan substrates for some of these enzymes are different enough from soluble starch that the real activity was not being measured.

The quadruple mutant lacking all of the BAMs except for BAM3 (*B3‐Q*) was phenotypically indistinguishable from WT plants in that it had no *sex* phenotype and was similar in size to WT plants at 5 weeks of age (Figures 1b and 2). This indicates that BAM3 alone is sufficient for normal starch degradation under the conditions used here. *B1‐Q* plants also grew normally, but had a partial sex phenotype intermediate between that of WT and *B‐Null* plants (Figures 1b and 2) indicating that BAM1 can replace the function of BAM3 in supplying enough night‐time sugars to support growth despite being unable to completely degrade leaf starch.

The ease with which mRNA levels can be measured in plants using microarrays and RNAseq has made these techniques widely used as a proxy for gene expression, but it is becoming widely recognized that despite large changes in mRNA levels either developmentally, diurnally, or in response to short‐term environmental stress, the levels of protein activity often remain relatively constant (Gibon et al., 2004; Piques et al., 2009; Skeffington et al., 2014; Vogel & Marcotte, 2013). Using the quadruple mutants *B1‐Q* and *B3‐Q,* we measured the activities of BAM1 and BAM3 as plants developed, over a 24‐hr photoperiod, and in response to several abiotic stresses, and compared our results with reported measurements of BAM1 and BAM3 mRNA. BAM1 activity in *B1‐Q* leaves was remarkably constant developmentally (Figure 5), diurnally (Figure 6), and it increased only marginally with osmotic stress (Figure 7a) despite large fluctuations in BAM1 mRNA over a diurnal period (Figure S3a) and in response to osmotic stress (Figure S4a). Mutants lacking BAM1 are compromised in osmotic stress‐induced starch degradation and water uptake resulting from decreased accumulation of osmolytes (Thalmann et al., 2016; Zanella et al., 2016), so this enzyme is clearly involved in these responses. Indeed, Monroe et al., (2014) observed compromised soluble sugar accumulation after two days of osmotic stress in plants lacking BAM1, but after four days, the plants lacking BAM1 accumulated as much reducing sugar as did plants with BAM1 so the response to osmotic stress is clearly complex (Monroe et al., 2014). Here, we also observed a large increase in soluble reducing sugars in *B1‐Q* plants over the treatment period (Figure 7b) suggesting that metabolism had changed dramatically over the 4 days of stress, but the increased sugar content is not likely a direct result of an increase in BAM1 activity alone. The contrasting effects of osmotic stress on BAM1 mRNA and activity levels suggest that there must be tight regulatory control over BAM1 activity, and this deserves further investigation.

Similarly, BAM3 activity in *B3‐Q* plants was relatively constant developmentally (Figure 5), and diurnally (Figure 6), despite large fluctuations of BAM3 mRNA (Figure S3b). These observations reinforce the need for a greater reliance on enzyme activity data or mass spectroscopy measurements of protein levels rather than mRNA levels to elucidate gene function. A role for BAM3 in the response of plants to cold stress was suggested by a strong increase in BAM3 mRNA after cold stress (Figure S4b; Kaplan & Guy, 2004, 2005; Monroe et al., 2014). Moreover, *bam3* mutants accumulated less sugar and were unable to maintain photosynthesis during cold stress (Kaplan & Guy, 2005). However, despite an increase in BAM3 mRNA during cold stress, BAM3 activity in *B3‐Q* declined by over 60% over several days at 4°C (Figure 8). We previously observed starch accumulation in cold‐stressed Arabidopsis leaves that could be due in part to the decline of BAM3 activity (Monroe et al., 2014). It was suggested that BAM3 protein might be post‐translationally inactivated by cold‐stress‐induced glutathionylation, which rapidly inactivates the enzyme with an adduct at C433 (Storm, Kohler, Berndsen, & Monroe, 2018). Alternatively, BAM3 may simply be rapidly degraded during cold stress. Indeed, Li et al. (2017) reported that BAM3 has a remarkable short half‐life of 0.43 days, which is among the shortest half‐lives of any Arabidopsis protein. The strong increase in BAM3 mRNA levels during the cold stress might enable a more rapid synthesis of BAM3 protein when the stress abates, facilitating recovery. Experiments using the *bam3* and *B3‐Q* mutants should be useful in addressing this question.

## 5 CONCLUSIONS

Using five quadruple mutants, each expressing only one of the five catalytically active Arabidopsis β‐amylases (BAMs) and a quintuple mutant lacking all five BAM genes, we show that in plants grown under a 12‐hr‐light/12‐hr‐dark photoperiod, complete leaf starch degradation comparable to WT, as detected by iodine staining, is dependent only on BAM3, but BAM1 alone can still provide enough carbon from starch degradation to support WT‐level plant growth. BAM2 and BAM6, despite their plastid location, do not contribute significantly to leaf starch degradation as detected by iodine staining, or to plant growth under the conditions tested, similar to the cytosolic BAM5. With these mutants, we can confirm that BAM activity in chloroplasts from WT leaves is mostly from BAM3, with some contribution from BAM1 and a trace from BAM2. Importantly, a plant without any of the five BAMs lacked any detectable BAM activity indicating that these BAMs are the only proteins with this type of catalytic activity present under these conditions. With these mutants, we were able to shed light on the isolated enzyme activity of BAM1 and BAM3 under different conditions to compare with previously reported transcript levels. Activity of BAM3 and BAM1 did not change diurnally, despite reports of relatively large changes in mRNA levels. BAM1 activity increased only marginally with osmotic stress, but not to the levels expected from increases in transcript levels. Likewise, BAM3 mRNA was reported to increase dramatically with cold stress, but the detected decline in BAM3 activity with cold stress suggests that cold‐induced starch accumulation may be partially a result of diminished BAM3 activity.

## CONFLICT OF INTEREST

The authors declare no conflict of interest associated with the work described in this manuscript.

## AUTHOR CONTRIBUTIONS

J.M. conceived the original research plans, performed the experiments, and wrote the article.
