# Supplementary material for: Involvement of five catalytically active Arabidopsis β‐amylases in leaf starch metabolism and plant growth
Source: Plant Direct. 2020 Feb 11;4(2):e00199. doi: 10.1002/pld3.199 (PMC7011640; doi:10.1002/pld3.199)
Supplement: Supplementary file 1 [file PLD3-4-e00199-s001.pptx]

## Slide 1
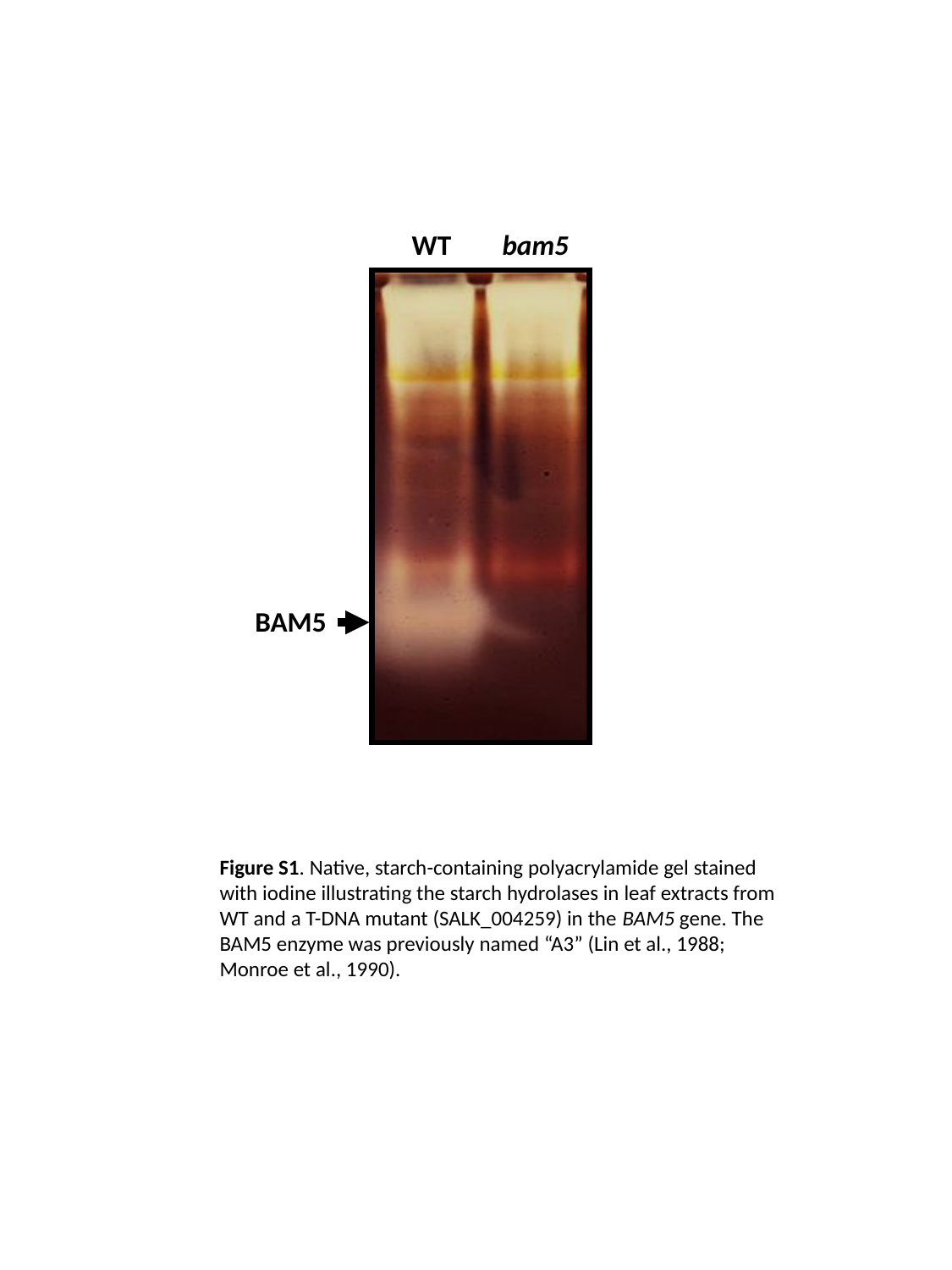

WT bam5
BAM5
Figure S1. Native, starch-containing polyacrylamide gel stained with iodine illustrating the starch hydrolases in leaf extracts from WT and a T-DNA mutant (SALK_004259) in the BAM5 gene. The BAM5 enzyme was previously named “A3” (Lin et al., 1988; Monroe et al., 1990).

## Slide 2
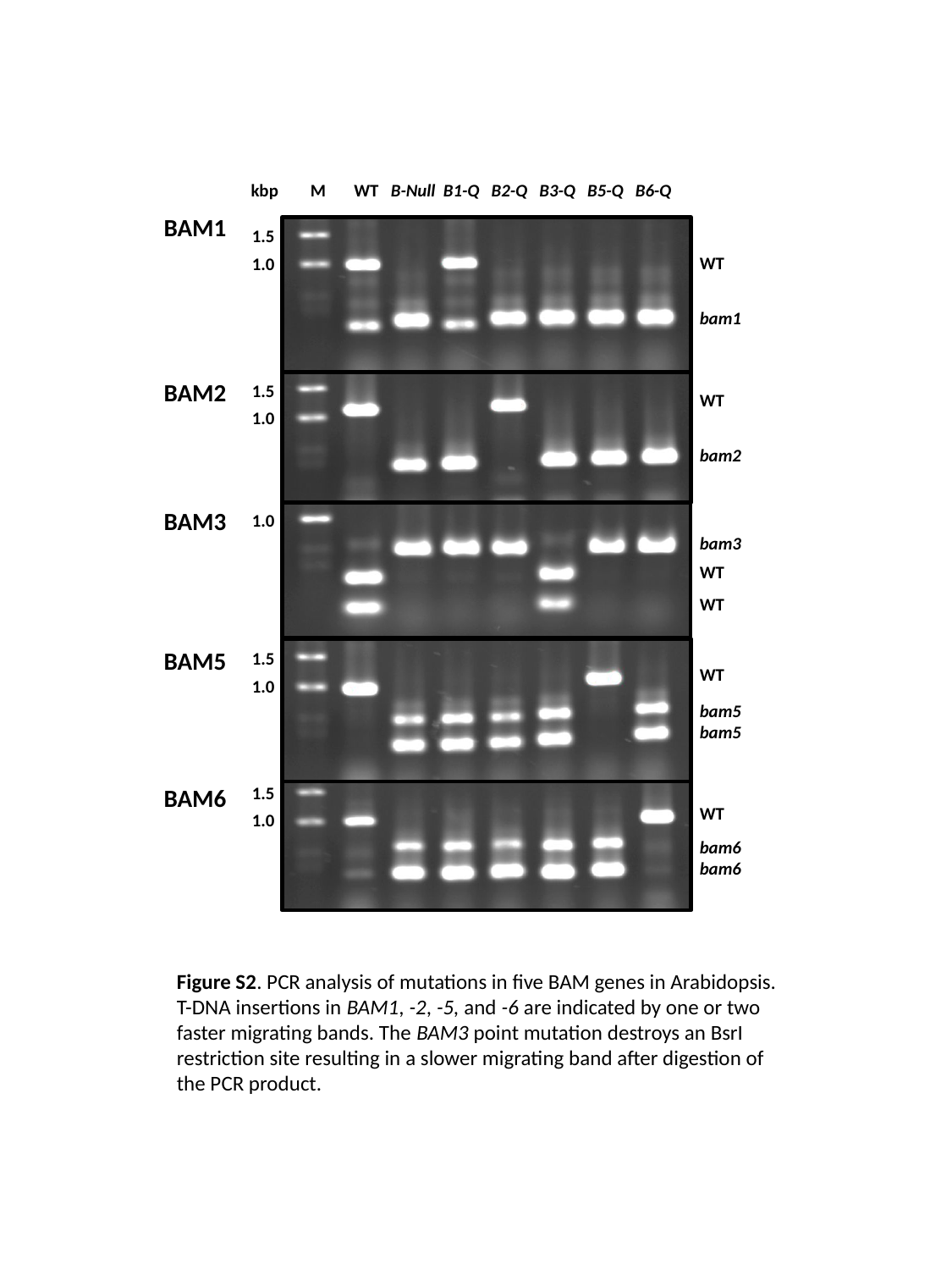

kbp M WT B-Null B1-Q B2-Q B3-Q B5-Q B6-Q
BAM1
1.5
1.0
WT
bam1
BAM2
1.5
1.0
WT
bam2
BAM3
1.0
bam3
WT
WT
BAM5
1.5
1.0
WT
bam5
bam5
1.5
1.0
BAM6
WT
bam6
bam6
Figure S2. PCR analysis of mutations in five BAM genes in Arabidopsis. T-DNA insertions in BAM1, -2, -5, and -6 are indicated by one or two faster migrating bands. The BAM3 point mutation destroys an BsrI restriction site resulting in a slower migrating band after digestion of the PCR product.

## Slide 3
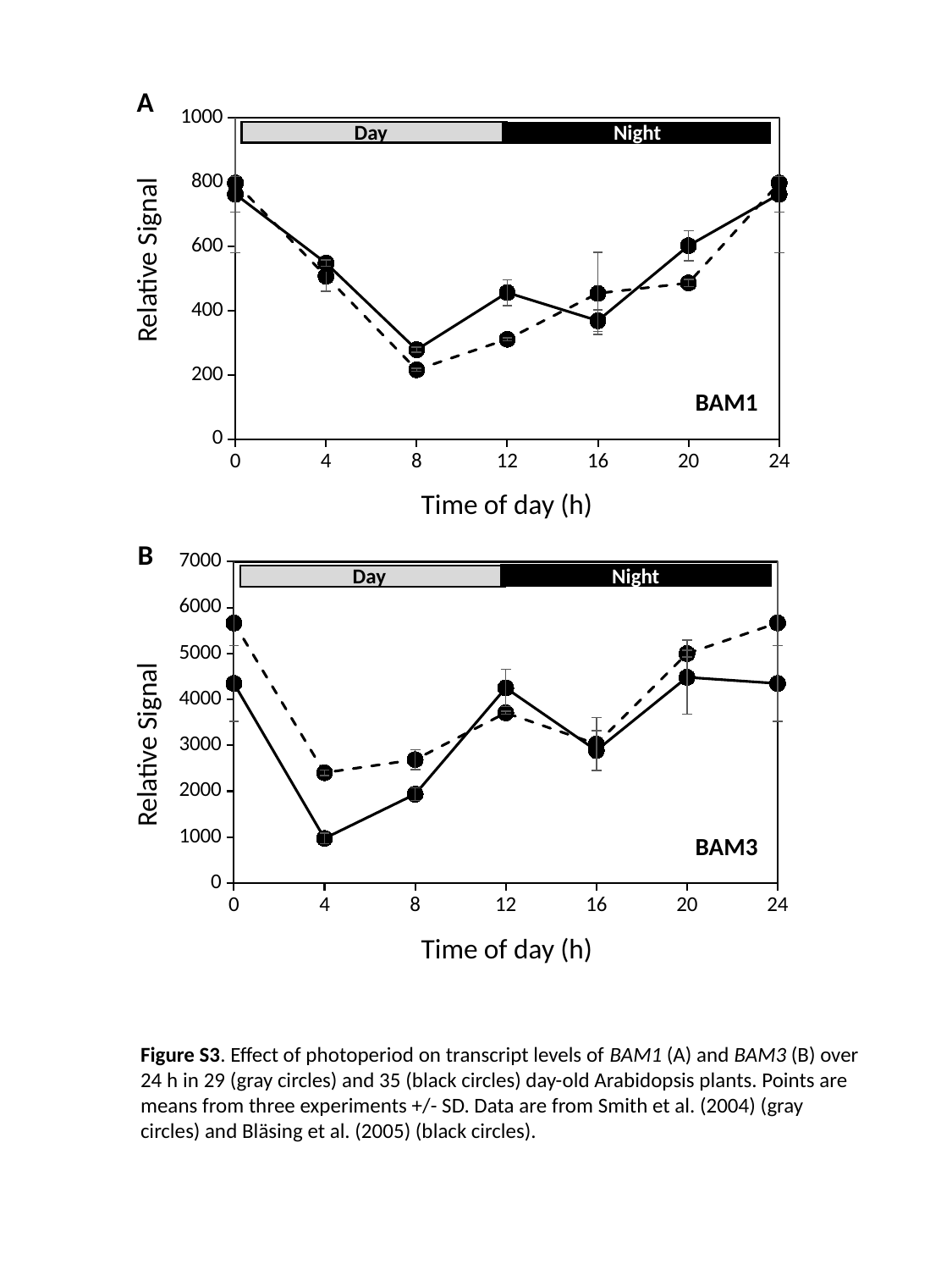

A
### Chart
| Category | | |
|---|---|---|Day
Night
Relative Signal
BAM1
Time of day (h)
B
### Chart
| Category | | |
|---|---|---|Day
Night
Relative Signal
BAM3
Time of day (h)
Figure S3. Effect of photoperiod on transcript levels of BAM1 (A) and BAM3 (B) over 24 h in 29 (gray circles) and 35 (black circles) day-old Arabidopsis plants. Points are means from three experiments +/- SD. Data are from Smith et al. (2004) (gray circles) and Bläsing et al. (2005) (black circles).

## Slide 4
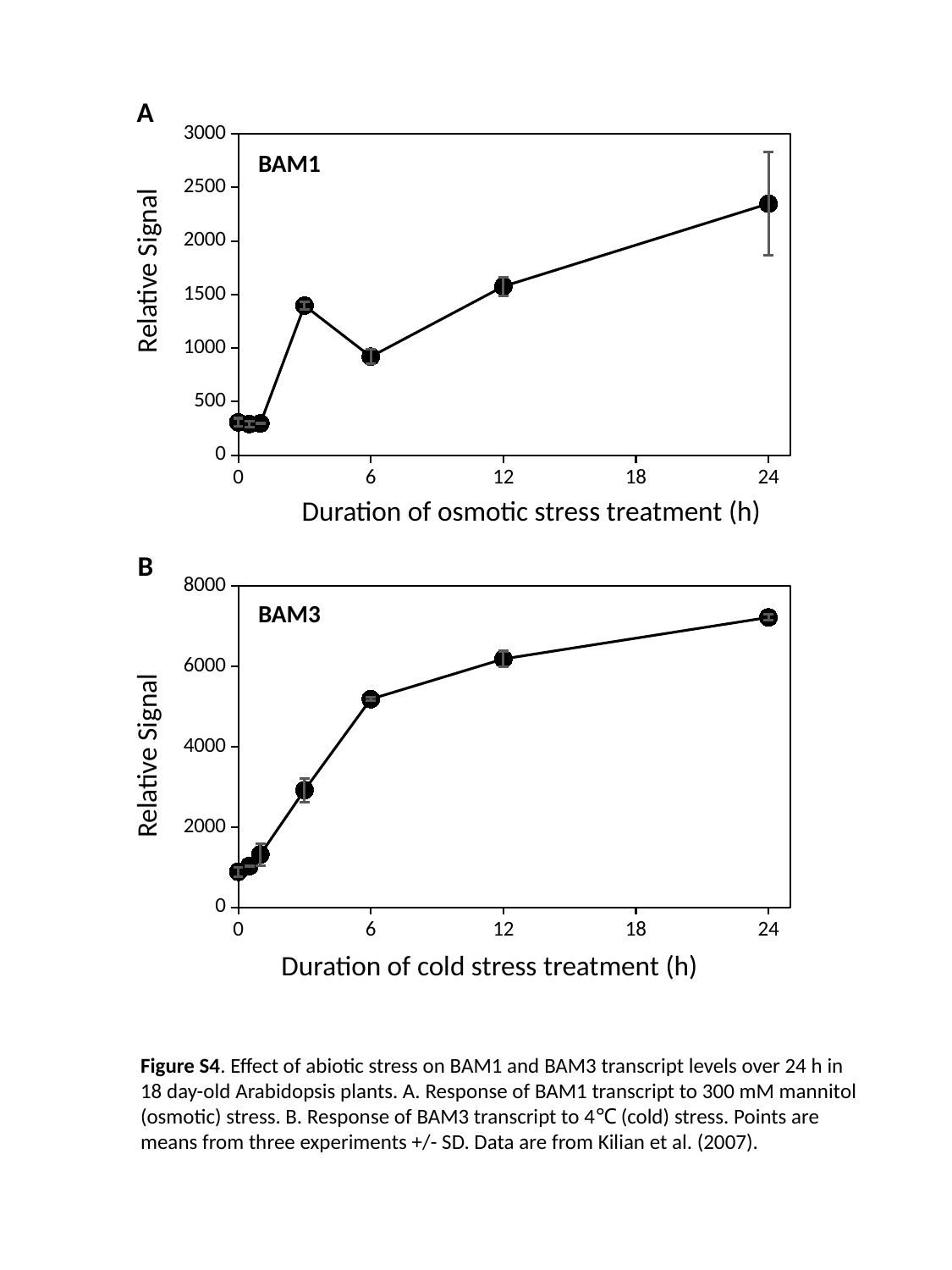

A
### Chart
| Category | |
|---|---|BAM1
Relative Signal
Duration of osmotic stress treatment (h)
B
### Chart
| Category | |
|---|---|BAM3
Relative Signal
Duration of cold stress treatment (h)
Figure S4. Effect of abiotic stress on BAM1 and BAM3 transcript levels over 24 h in 18 day-old Arabidopsis plants. A. Response of BAM1 transcript to 300 mM mannitol (osmotic) stress. B. Response of BAM3 transcript to 4℃ (cold) stress. Points are means from three experiments +/- SD. Data are from Kilian et al. (2007).
