# Supplementary figures and images for: Involvement of five catalytically active Arabidopsis β‐amylases in leaf starch metabolism and plant growth
Source: Plant Direct. 2020 Feb 11;4(2):e00199. doi: 10.1002/pld3.199 (PMC7011640; doi:10.1002/pld3.199)

## Slide 1
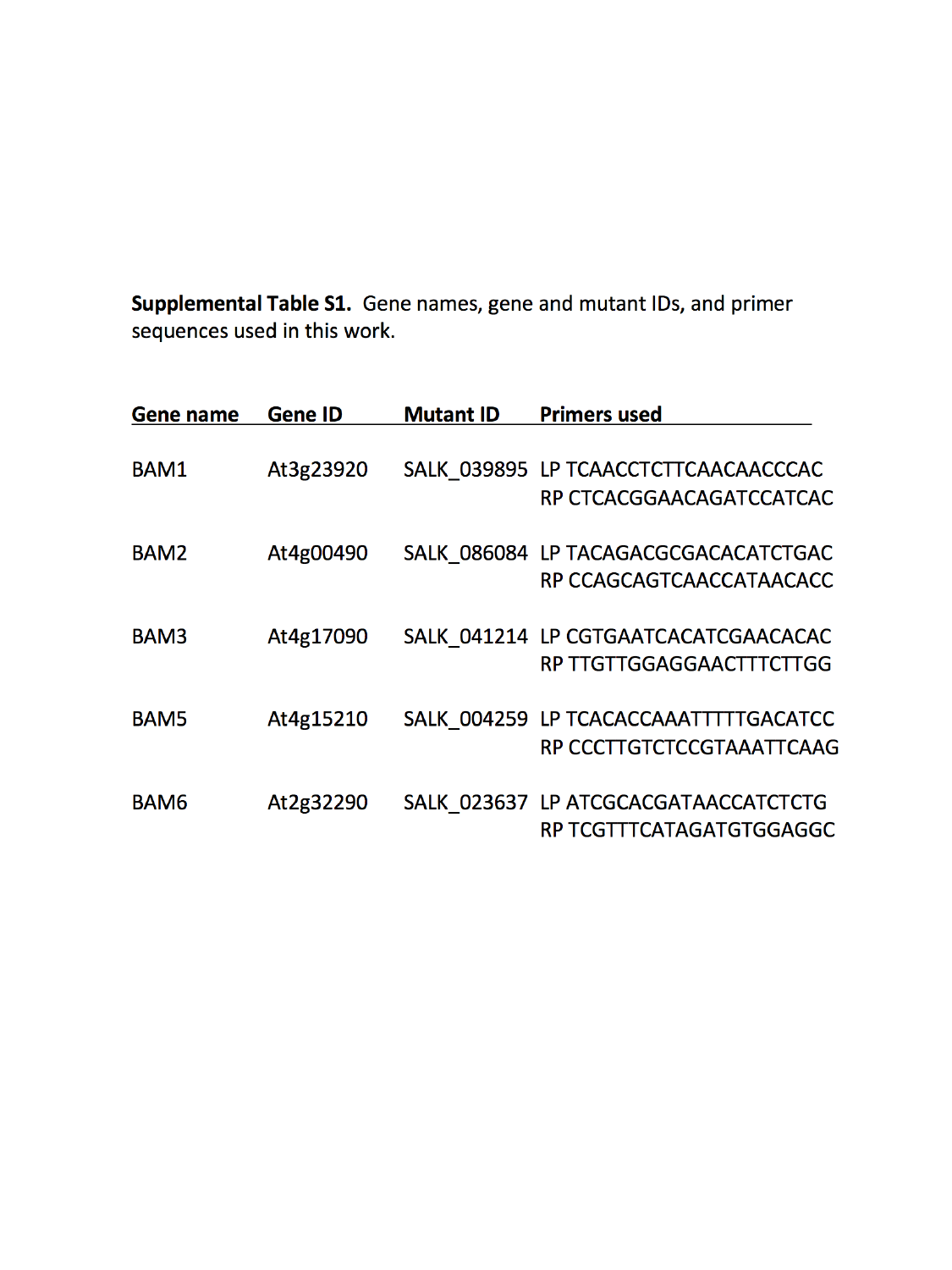

Supplement: Supplementary file 2 [file PLD3-4-e00199-s002.pptx]
